# Beginning a new medical school curriculum amidst a global pandemic

**DOI:** 10.1096/fba.2020-00097

**Published:** 2020-12-22

**Authors:** Nirav G. Shah, Devang M. Patel, Norman F. Retener, Philip C. Dittmar, Constance Lacap, Kerri A. Thom, Joseph Martinez

**Affiliations:** ^1^ Department of Medicine University of Maryland School of Medicine Baltimore MD USA; ^2^ Department of Psychiatry University of Maryland School of Medicine Baltimore MD USA; ^3^ Department of Epidemiology & Public Health University of Maryland School of Medicine Baltimore MD USA; ^4^ Department of Emergency Medicine University of Maryland School of Medicine Baltimore MD USA

**Keywords:** collaborative learning, COVID‐19, curriculum, medical education

## Abstract

The University of Maryland School of Medicine embarked on our first major curriculum revision since 1994 with a plan to implement this Renaissance Curriculum in August 2020. However, in the Spring of 2020, the coronavirus disease (COVID‐19) pandemic disrupted clinical care and medical education on a large scale requiring expeditious modifications to our Renaissance Curriculum as well as our traditional Legacy Curriculum in order to meet our goal of educating the next‐generation of physicians. The rippling effects of the COVID‐19 pandemic led to major changes in the delivery of the pre‐clerkship curriculum, the way we assessed and evaluated students, entry into the clinical environment, length of clinical rotations, and orientation for our new medical students. We relied on “new” technology, digital medical resources, and the creativity of our educators to ensure that our learners continue to acquire the skills necessary to become skilled clinicians in these unprecedented times.

## 1 INTRODUCTION

In June 2018, the medical education leadership at the University of Maryland School of Medicine (UMSOM) embarked on our first major curriculum revision since 1994. In preparation, our methodology included speaking with other institutions, studying curricula implemented across the country, and identifying best practices. At the same time, we recognized the importance of identifying the environment and values that contribute to making UMSOM excellent in biomedical education, basic and clinical research, and quality patient care and service. Our new curriculum, the Renaissance Curriculum, was on target to be implemented in August 2020.

In the Spring of 2020, the coronavirus disease (COVID‐19) pandemic caused by SARS‐CoV‐2 massively disrupted both clinical care and medical education. In this manuscript, we describe the major changes we quickly, but thoughtfully, implemented to meet the needs of our learners in our Legacy Curriculum and the changes that we instituted as we launched our state‐of‐the‐art Renaissance Curriculum in order to balance our learner's education with the responsibility to protect their health and safety.

## 2 SPRING 2020: NEW COVID‐19 COURSE

Given that the COVID‐19 pandemic will be one of the most meaningful events in our medical students' careers we determined we needed a COVID‐19 course to highlight the biomedical sciences, the clinical manifestations, the effect of health disparities, and the psychosocial impact on the lives of patients, staff, and healthcare professionals. After quickly recognizing the challenges with United States Medical Licensing Examination (USMLE) testing at Prometric centers and the rapidly evolving situation surrounding cancellations, we made the decision to move our normal window for USMLE Step 1 testing from May and June to August. This change enabled us to implement a COVID‐19 course for our rising third‐year students prior to taking USMLE Step 1 and subsequently entering the clinical phase of their education. This course was mandatory for the Class of 2022 and available as an elective for any member of the other medical school classes.

**FIGURE 1:**
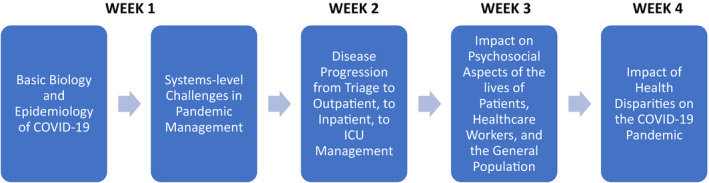
Timeline and general content themes of Spring 2020 new COVID‐19 course

We felt it imperative to create a curriculum that encompassed the epidemiology and public health response, the pathophysiology, diagnosis, treatment, and prevention of the disease. To that end, we created a course entitled “COVID‐19: From the Bench to the Bedside” to help our rising second‐year, third‐year, and fourth‐year students understand the basic science of the disease process and prepare them to enter the clinical environment knowledgeable about COVID‐19 and equipped with the clinical skills necessary to function in a rapidly evolving health care system (Figure 1).

Attendance was mandatory for the rising third‐year students, and there were daily assignments and quizzes, as well as a required narrative reflection to be submitted at the completion of the course. The feedback and review for this course was overwhelmingly positive. The students particularly valued the discussions about Ethics, Health Policy and Health Disparities, as well as the virtual clinical rounds. At the completion of this course, the Class of 2022 entered their dedicated period to study and prepare for the USMLE Step 1 examination, and we turned our attention toward the Class of 2024 that would begin the Renaissance Curriculum with Introduction to Medical School in August 2020 and the Class of 2021 that was reentering the clinical arena.

## 3 FALL 2020: INTRODUCTION TO MEDICAL SCHOOL

Our Introduction to Medical School (IMS) for M1's is typically three days in duration, held in‐person, and accomplishes the goals of orienting the students to life in medical school, introducing incoming students to various services within the school and campus and allowing student‐student and student‐faculty interaction. Under the Renaissance Curriculum, we planned to completely revamp the IMS curriculum and expand this course to an entire week, culminating in our White Coat Ceremony. This year, the COVID‐19 pandemic precluded these plans. Instead, we held the entire week‐long course over Zoom, and we deferred the White Coat Ceremony to a later date.

Under the pandemic constraints, it was even more important to have IMS feel cohesive, fostering meaningful interactions between incoming students and those from classes ahead of them as well as with faculty members. Ultimately, the course was also informed heavily by the events surrounding the death of George Floyd just weeks before, and the need for the School to be thoughtful and comprehensive in its response. The course was organized around daily themes (Table 1).

**TABLE 1:** Daily themes for introduction to medical school

Monday	“Meet and Greet”
Tuesday	“Curriculum Day”
Wednesday	“Becoming a Part of our Community”
Thursday	“How to Be a Clinician”
Friday	“Wellness/Life on Campus”

Several notable sessions are worth describing. As part of the “Becoming a Part of our Community” day, the students began with a didactic lecture describing the history of structural racism in Baltimore and how it continues to impact the health of our patients today. The students were then taken on a virtual tour of Baltimore by one of our faculty members who traditionally gives the tour in‐person on a chartered bus. This year, he recorded the tour on his cell phone from his car. He then narrated the tour for the students and added additional discussion points synchronously via Zoom. A lecture on social determinants of health was then followed by breakout rooms led by near‐peer students. These students had met with faculty members to discuss the format and content of the sessions, but the sessions themselves were student‐only. An introductory lecture about LGBTQI+ health was then supplemented by a vibrant panel discussion about issues surrounding the health of this population. All students subsequently underwent Unconscious Bias training.

One of our most unique, powerful, and best regarded sessions was on “How to Be a Clinician” day. The students participated in “COVID rounds” in the Medical Intensive Care Unit (MICU) of the University of Maryland Medical Center. Our Assistant Dean for Curriculum, who is a Pulmonary/Critical Care physician, and our Director of Pre‐clerkship Curriculum, who is an Infectious Disease specialist, took the students on virtual rounds in the MICU, speaking directly with teams caring for critically ill patients infected with COVID‐19. This was done over a HIPAA‐compliant Zoom platform, utilizing an iPad. Other physicians on the education leadership team curated the Chat Box, providing real time information about medical terminology. Students were then allowed to debrief at the end of the session and ask further questions. Evaluations for this session were uniformly positive, with such comments as, “I thought the sessions on the university's response to COVID were engaging and very thorough. I really appreciated the openness of the faculty in describing their experiences with treating the disease and addressing the information being distributed in mainstream media. The rounds were spectacular and inspirational. The fact that Dr. [X] and Dr. [Y] walked us through the entire ward and engaged with other team members and asked them questions about current patients was really meaningful.” “The COVID rounds was my favorite session today. Hearing about a patient case reminded me of how exciting medicine will be.” “I think the COVID Rounds was my favorite part of Orientation so far! It made me excited to become a physician.”

Lessons learned from the first week of remote instruction were that breaks need to be even longer and more frequent than planned, even the most engaging lectures should have interactive components, and early exposure to real‐life clinical medicine is incredibly valuable and sets the right tone for the start of a student's medical career. This week‐long introduction was followed by the beginning of the first course of 2020 for both M1's and M2's.

## 4 PRE‐CLERKSHIP CURRICULUM

The pedagogical approach in the pre‐clerkship curriculum was developed to be a combination of lectures, small‐group discussion, laboratory sessions, and clinical correlates centered around a patient and/or family affected by a disease process. It was important to maintain this structure through a combination of synchronous and asynchronous teaching methodologies. We utilized the Zoom platform for videoconferencing given that it was easy‐to‐use, mobile‐friendly, and faculty members at the University of Maryland, Baltimore (UMB) are provided with institutional accounts with the ability to host online meetings with up to 300 participants. To augment the pre‐clerkship curriculum, we investigated multiple medical learning platforms and ultimately chose AMBOSS to supplement the learners' didactic curriculum with a digital medical resource that included a comprehensive library as well as a robust question bank. In addition, in the Renaissance Curriculum, we created primers to ensure that our learners had the foundational knowledge prior to participation in the coursework for any given topic. The Renaissance Curriculum is focused on increasing the amount of active learning and improving the quality of the educational material delivered to our students. Given the need to deliver the content remotely, we mandated that most lectures have audience response questions incorporated as well as multiple breaks for small group discussion. This allows the diversity of our medical students to help facilitate a rich dialog and aid in the comprehension of the material being taught.

We chose synchronous delivery of M1 and M2 lectures over Zoom to allow real‐time engagement of students and faculty. This came with some technical hurdles with faculty having various degrees of comfort using Zoom. Our preferred method of lecture delivery is via Zoom from one of our lecture halls where we have support from technology specialists. Similar to pre‐pandemic, faculty deliver their content in the lecture hall. However, unlike past years, the lecture hall is empty, and students are engaged through a ClearTouch board (a large, interactive LCD display with integrated PC and WiFi module) set up in front of the lecturer. The lecturer can see students through Zoom and can view comments on this large board as they lecture from the podium. We asked all lecturers to incorporate questions using TurningPoint polling software in order to gauge students' understanding of the content and to keep them engaged with the didactic material.

One of our biggest challenges was with the anatomical sciences section of our Foundations course. We made the difficult decision early on that in‐person anatomic dissection with groups of students clustered in close proximity around cadavers was unsafe. Utilizing lessons learned from our Neuroscience course in April, just after the pandemic forced cessation of in‐person instruction, we devised a plan of guided video dissection. Our faculty member who serves as content lead for anatomy, along with a trauma surgeon, dissect over Zoom utilizing multiple high‐definition cameras affording different views including wide‐angle and close‐up. Their discussion while dissecting often proved to be highly beneficial for the students to learn clinical scenarios that correlated with the area of dissection. Students then enter breakout rooms where they repeat the dissection using 3D software called ZygoteBody. The groups upload a screen capture of their completed project for course director review. The lab session involved multiple clinical faculty members to assist in the dissections and provide further clinical correlation to the students. In addition, interactive clinical cases are utilized during the dissection using TurningPoint polling software and Zoom breakout rooms.

## 5 OVERARCHING PRINCIPLES FOR THE RENAISSANCE CURRICULUM

### 5.1 Collaborative learning

The Renaissance Curriculum emphasizes active learning, teamwork and problem‐solving. Collaborative learning in a fully remote environment carries significant obstacles. Utilizing breakout rooms over Zoom has helped to divide our class into smaller groups, allowing for more collaborative work. The size of these groups can vary from very small groups of 3–5 students to larger groups of 20–30 students. Pre‐assigning breakout rooms ahead of time can decrease the downtime during a session and reduce the amount of technological challenge in that session. However, the work to create these pre‐assigned groups can be significant on the front end. In addition, once the students have broken into these groups during a session, when they return to the large group, it is not possible to have a second set of different breakout rooms pre‐assigned. The only options are to re‐use the same groups for the duration of the session, to manually assign breakout rooms, or to randomly assign rooms based upon evenly splitting the participants into a preset number of rooms. This is not difficult from the vantage of dividing the learners, but to then ensure that a facilitator is present in each room carries some challenges.

Although we successfully incorporated collaborative learning into the distanced‐educational model, we believe that in‐person learning has specific benefits in both development of collaborative skills as well as for the assessment of those skills. As such, we collaborated with school and campus officials, epidemiologists, infection preventionists, and facilities and operations management teams to deliver small in‐person sessions safely under pandemic restrictions. Students and faculty received COVID‐specific education, underwent COVID testing according to campus guidelines and participating in a daily symptom monitoring program. School policies were updated liberalizing absenteeism and emphasizing participation in public health strategies including reporting of symptoms and contact tracing. The in‐person sessions focused on cases and problems to be discussed in small groups with an emphasis on team‐work and use of a white board to help our adult learners learn from each other under the guidance of a faculty preceptor. These sessions have been well‐received for their content and their secondary goal of building community and camaraderie.

#### 5.1.1 Collaborative learning space

Prior to the pandemic, we had begun the process of renovating our existing learning spaces. These multi‐disciplinary labs were a holdover from the 1990s. They were small rooms and were configured to support a time when learning consisted of a centrally located educator in each room with learners at the perimeter of the space, peering into microscopes with their backs to the preceptor. Current students would often either sit with their laptops on their laps in order to face the preceptor, or sit with their laptops on the counters, forcing them to have their backs to the preceptors. Thanks to a generous gift from an alumnus, we were able to renovate eight of these rooms into two large spaces with round tables for the students to sit at, wireless monitors on the perimeter walls for educational materials to be casted to, and ample space for facilitators to walk around to different groups of learners. These rooms were built to hold approximately 80–90 students each.

With the COVID‐19 outbreak, these rooms have not been used to full capacity or capability yet. We have begun to bring learners back in small numbers to this environment, with 18–20 learners per room, all masked and physically distanced within the room. Cleaning stations are located in each room for learners use to clean their personal workstation before and after each session. The rooms are then thoroughly cleaned each day by environmental services.

#### 5.1.2 In‐person vs Zoom

While the breakout room feature of Zoom is very useful to allow collaborative learning to occur, we have found that in‐person discussion groups allow for more vibrant interaction with more in‐depth discussion. There were many obstacles to overcome, from COVID screening, to providing a safe environment, to ensuring students and faculty feel comfortable participating in person under a pandemic. Many of our students were happy to resume in‐person activities; however, some students have personal reasons for opting out of in‐person learning, including reasons related to personal health risks or those with whom they share housing. Students were thereby given an option to select either the hybrid, in‐person and distanced learning described above, or to fully participate in distance learning only, without in‐person sessions. Students may select either option without cause or individual repercussion. Students were advised, however, that certain aspects of the curriculum, e.g., clinical skills training as part of the M1/M2 longitudinal clinical curriculum (described below in more detail), are not conducive to distanced learning. In this case, students would be subject to remediation, if possible, using a combination of simulation and real‐patient experiences at a later time, to meet core curricular requirements. Very few students chose to opt out of in‐person instruction.

#### 5.1.3 Team‐based learning

Team‐based learning (TBL) plays a large part in our Renaissance Curriculum. During Introduction to Medical School, we introduced the concept and held a straightforward TBL session to familiarize the students with this pedagogy. We utilized Microsoft Forms during the session, which highlighted some technical difficulties. We then made the decision to invest in commercial TBL software (InteDashboard) to streamline the process. We had also envisioned running parallel sessions as part of our hybrid model, with two TBL preceptors in‐person and several others on Zoom. Given the logistical challenges of trying to integrate and facilitate discussion between the students on Zoom and the students in the in‐person sessions, we decided to hold our TBL sessions over Zoom, in their entirety. We also incorporated TBL into our Legacy Curriculum for M2's. This was well‐received by these students who then asked the education leadership team for more of this style of learning. This led to course leadership adjusting a planned smaller group session into a TBL.

## 6 CLINICAL ROTATIONS

The clerkship curriculum is singularly focused on live, in‐person clinical experiences with supplemental didactics to enhance the clinical education. The pause in clinical rotations due to COVID‐19 occurred three‐quarters of the way through the clerkship year, meaning all students had at least one core clerkship to complete as they entered their fourth year. The decision to resume clinical rotations came as a result of improving local COVID‐19 infections rates, greater availability of personal protective equipment, and the development of tracking and reporting applications at the campus level.

### 6.1 4th year reentry to clinical rotations

The return date for our rising fourth‐year students aligned with the start of our traditional academic year. In order to return to clinical rotations, each student was required to complete standardized training related to the changing patient care landscape. This training included reviewing recorded lectures from our COVID‐19 course on COVID‐19 transmission dynamics and infection prevention strategies, student Health safety information, safety reporting mechanisms at our two major clinical sites, and personal protective equipment donning and doffing videos. The students were then required to pass an assessment based on the materials. Additionally, each student also had to undergo N95 fit testing.

Prior to the pandemic, the clerkships were focused on expanding clinical sites to accommodate higher numbers of students as a result of the shortened pre‐clerkship curriculum with the coming Renaissance Curriculum. Unfortunately, many of these plans were in the early stages and newly added sites were reluctant to resume clinical rotations on the same schedule as UMSOM. This strain was most apparent with ambulatory sites as many outpatient clinics moved to telehealth visits for some or all their patient visits and others did not have an adequate supply of personal protective equipment. Additionally, there were challenges with rescheduling missed clerkships due to variable clerkship length at 4‐weeks, 6‐weeks, or 8‐weeks in duration. We shortened two clerkships from 6‐weeks to 4‐weeks for this academic year to allow for greater flexibility with scheduling and rescheduling of student clerkships. Of particular note, despite shortening the length of clerkships, we did not alter the performance standards required to successfully complete a clerkship. Scheduling was further complicated by students' conflicting scheduling goals due to a desire to quickly complete their core clerkships and also complete career‐specific electives and sub‐internships prior to the start of the residency application season. The Office of Student Affairs met with each student to discuss their scheduling goals and an individual schedule was built to meet each student's priorities and graduation requirements.

### 6.2 3rd year entry to clinical rotations

The return of the rising third‐year students presented a varied challenge due to complexities with their entry and exit from the clerkship year due to USMLE Step 1 scheduling on one end and schedule preparations for the Renaissance Curriculum on the other end. As previously mentioned, the students were unable to reliably schedule their USMLE Step 1 examination dates in May and June, and therefore, a decision was made to shift all USMLE Step 1 examinations to August. The students then completed the COVID‐19 course in June which culminated in the Introduction to the Clinical Years sessions and Student Clinician Ceremony. In a typical year, the Introduction to the Clinical Years and Student Clinician Ceremony occur the week prior to the start of clerkships and consist of in‐person large group, small group, near‐peer, and hands‐on procedural simulation sessions with the class ceremony on the final day. Due to the pandemic, the sessions that could be delivered in a virtual format were run synchronously via Zoom and the sessions that required in‐person sessions were removed. Similar to the rising fourth‐year students, the rising third‐year students were required to pass an assessment based on the materials presented during these sessions. The students then entered a dedicated period to study and prepare for the USMLE Step 1 examination.

Moving from a July start to a late August start for the clerkship year meant that we needed to shorten our clerkship year so that the following class could start on schedule for the next academic year. Our 48‐week Legacy Curriculum clerkship year consists of 40 weeks of core clerkships and 8 weeks of electives. To achieve a 40‐week clerkship year, an easy solution would be to drop the 8 weeks of elective rotations, however, our variable clerkship length would not allow streamlined transitions between clerkships due to our winter break no longer falling at the midpoint of the academic year. Therefore, the 40‐week clerkship year was accomplished by shortening the duration of the OB/Gyn and Pediatrics clerkships from 6‐weeks to 4‐weeks and losing one of two 4‐week clinical electives.

### 6.3 Physical distancing

The changes to the clinical environment due to COVID‐19 affected many aspects of our clinical education. The leadership from the UMB campus and the University of Maryland Medical Center (UMMC) adopted a policy where students were not permitted to interact with any COVID‐19 positive patients or persons under investigation for COVID‐19. This limited the pool of patients that could be seen by student learners. COVID‐19 also affected how we run teams and how we teach. Many team rooms or work rooms were identified as having insufficient space for adequate physical distancing. As a result, new workspaces were identified, and remote conferencing options were utilized to allow for physical distancing. In addition, clerkship didactics could no longer be held in‐person due to UMSOM policy, so these didactics were moved to virtual sessions via Zoom. Clerkship directors were encouraged to dismiss students for the day during didactics to allow students to participate remotely from outside the hospital and thus, decompress the workspaces.

### 6.4 Safety measures

The safety of our students was a steadfast goal as we resumed clinical rotations. The UMB campus developed online tracking and reporting applications for all students, faculty and staff. Additionally, the UMB campus developed a hotline for students in case of an exposure to COVID‐19. Students were trained on safe donning and doffing techniques by UMMC Infection Control, and the medical education leadership adjusted our guidance for students based on a few months of clinical experience in the absence of students and the information gained immediately following the return of students.

## 7 LONGITUDINAL CURRICULUM

The longitudinal curriculum is focused primarily on clinical skills teaching (history taking, physical examination and medical decision making), and also covers topics important for our students to become well‐rounded physicians and citizens (social justice/health inequalities, service learning, health care policy, quality improvement, and nutrition).

### 7.1 Clinical skills

Teaching clinical skills in the COVID environment proved to be one of the most challenging problems we had to navigate. Our clinical skills courses include in‐person preceptor sessions where students interview and examine patients in the clinical space under the supervision of a faculty member as well as virtual encounters in our Standardized Patient Laboratory where students are able to practice and learn the history and physical from patient actors.

For our in‐person preceptor clinical sessions, we collaborated with experts from campus to allow this to continue in small groups. Working with the Office of Medical Education, we used our House Advisory System virtual houses as our cohorts to plan all small group activities. We also limited these groups to five persons or less, while providing students with facemasks and full face‐shields. These measures will allow us to limit potential exposures while also allowing for streamlined contact tracing in the event of any positive COVID tests. In accordance with SOM policy, students will not be permitted to interact with COVID‐positive patients or persons under investigation for COVID. Additionally, we relaxed our professional dress code to allow students to wear scrubs in the clinical space for ease of laundering.

For our Standardized Patient encounters we flexed as many of these to virtual as possible. All communication‐based encounters were made into virtual “tele‐health” encounters that the students could participate remotely. These were supplemented with prepared recorded examples of a patient interview. For our physical examination simulated encounters, students were given the opportunity to meet with our Physical Exam Teaching Associates virtually to learn different portions of the examination and allow them to practice the examination in real time with a classmate or roommate volunteer, while receiving feedback from the Teaching Associates.

### 7.2 “The well‐rounded physician”

Our Service‐Learning Program also proved challenging to implement under the constraints of a pandemic. Our pre‐clerkship students are required to do 30–40 h participating with one of our community partners (which focus on Baltimore City Public School (BCPS) programs). With the onset of COVID, all students were removed from their programs as all in‐person activities were suspended. Some of our students were able to continue their work virtually. Working with our community partners, we were able to expand this and move most of our programs into the virtual space. Our students will be participating with these programs through virtual mentoring with BCPS students. We found that this has opened more opportunities for our students to connect with the community as they are no longer bound by meeting in a physical space.

Our nutrition curriculum prior to COVID included in‐person cooking sessions in our integrative medicine center. Students would receive a didactic session, then they would cook and eat together in the center's test kitchen. This was replaced with video recordings of the lectures and cooking sessions, and the recipes were altered so that the students would be able to try these recipes at home. Additionally, Q&A sessions were held over Zoom following the release of educational material.

The remainder of our didactic sessions have operated much in the same way as the rest of the curriculum, with real time lectures given over Zoom and 25% of the students participating in the small groups in‐person, while the rest participate in the virtual space. We have also included technologies such as Turning Point to make our didactic sessions more interactive. We are continuing to build more online and virtual content for our students to cover many of these topics, including a virtual “poverty‐simulation.”

## 8 ASSESSMENTS AND EVALUATIONS

### 8.1 Assessments

Due to COVID‐19, assessments in the Renaissance Curriculum have been remotely administered using the ExamSoft platform. Despite being administered remotely, in the students' own space, our School's Honor Code remains in effect. Students sign an attestation prior to the assessment stating that their behavior will align with the school's Honor Code.

In order to ensure that students are prepared to take an exam at home, we were in constant communication with them regarding device requirements, and internet capabilities. Our curriculum support staff have assisted students in troubleshooting when necessary. Approximately, 2 weeks prior to each assessment, we survey the class regarding any current technology issues, and follow up with individuals who may need to take the assessment in the medical school lecture hall with appropriate masking and social distancing. These students must comply with all campus COVID‐19 testing requirements.

All assessments are reviewed by our Assessment Review Committee (ARC) prior to being given to students. The ARC is comprised of Assistant/Associate Deans in the Office of Medical Education, course directors, content leads and other senior faculty well‐versed in item‐writing. Assessment items are collected from course instructors and the course directors, with a goal of having all items approximately two weeks prior to the assessment. The ARC then has pre‐work to complete prior to the meeting. The ARC meets via Zoom due to COVID‐19, and suggested edits are discussed in the larger group and finalized. Once the assessment has been given, statistical analysis is completed on each question. The ARC then has a Post‐Assessment meeting, in order to review the performance of the assessment overall, as well as individual items. This provides feedback to the course leadership in order to make improvements for future assessments within the course and in future years. In addition, this post‐assessment allows for identification of concepts that may have been unclearly taught, which allows the course director to review these with the students.

### 8.2 Evaluations

At the start of the Renaissance Curriculum, it was very important for us to obtain as much real‐time feedback from students as possible. This would allow Office of Medical Education (OME) to make mid‐course corrections where necessary. We again utilized our House Advisory System which naturally divides our MS1 class into four equal groups. We developed a rotation whereby each week one House was responsible for giving feedback on all educational sessions (lectures, small group sessions, TBL session, clinical correlates, labs/dissections). At the end of that week, a subset of students in that House are responsible for attending a focus group with faculty and OME Deans. Due to COVID‐19, our focus groups are held via Zoom. During the focus group, students have an opportunity to review the week's educational sessions and offer suggestions for improvement or discuss aspects of content delivery that they found especially effective. With this new rotational system, students are free to provide feedback on any sessions in their off weeks but are not required to do so. This decreases the overall burden of the evaluation process. Additionally, it appears that we are getting higher quality feedback as students are not waiting until the end of the course to evaluate individual lectures and sessions.

The session feedback is collected on our learning management system (MedScope). Students can rate the instructor effectiveness, the teaching materials, as well as the overall session. They are able to provide anonymous written comments that are available for course leadership to view, thus, allowing for more timely feedback to lecturers and ability to make real‐time changes as we continue through the curriculum.

## 9 CONCLUSION

The last 6 months at the University of Maryland School of Medicine have been challenging, rewarding, exciting, exhausting, and unforgettable. Our faculty, staff, and students have been nimble, flexible, and patient as we launched our Renaissance Curriculum, maintained our Legacy Curriculum, and navigated the COVID‐19 pandemic. We are confident the changes we made to our curricula will result in graduates of UMSOM that understand what it takes and what it means to be a physician reflecting our mission statement of creating lifelong learners who are clinically excellent, possess humanism, professionalism, scholarship, leadership, critical thinking skills and attention to social justice and diversity.

## AUTHOR CONTRIBUTIONS

N. Shah and J. Martinez designed the manuscript and N. Shah, D. Patel, N. Retener, P. Dittmar, C. Lacap, K. Thom, and J. Martinez wrote the manuscript.

